# Worldwide view of nephropathic cystinosis: results from a survey from 30 countries

**DOI:** 10.1186/s12882-017-0633-3

**Published:** 2017-07-03

**Authors:** Aurélia Bertholet-Thomas, Julien Berthiller, Velibor Tasic, Behrouz Kassai, Hasan Otukesh, Marcella Greco, Jochen Ehrich, Rejane de Paula Bernardes, Georges Deschênes, Sally-Ann Hulton, Michel Fischbach, Kenza Soulami, Bassam Saeed, Ehsan Valavi, Carlos Jose Cobenas, Bülent Hacihamdioglu, Gabrielle Weiler, Pierre Cochat, Justine Bacchetta

**Affiliations:** 1 Centre de référence des maladies rénales rares Néphrogones, hôpital Femme–Mère–Enfant, Hospices Civils de Lyon & Université Claude-Bernard Lyon 1, Lyon, France; 2 Hospices Civils de Lyon, Pôle Information Médicale Evaluation Recherche, Lyon, France; 3 Epidémiologie, Pharmacologie, Investigation Clinique CIC 1407 Inserm, Information médicale, Mère-Enfant–Bron, Bron, France; 4 Université Lyon 1, Equipe d’Accueil 4129, Lyon, France; 5 Medical School, University Children’s Hospital, Skopje, Macedonia; 6 Ali Asghar children Hospital, Teheran, Iran; 7 Division of Nephrology and Dialysis, Children’s Hospital Bambino Gesù, IRCCS, Roma, Italy; 8 Children’s Hospital, Hannover Medical School, Hannover, Germany; 9 Clinica Nefrokids, Curitiba, Brazil; 10 Service de néphrologie pédiatrique, Hôpital Robert Debré, Université Sorbonne Paris, Paris, France; 11 Birmingham Children’s Hospital, Birmingham, England; 12 Service de néphrologie pédiatrique, Hôpital Hautepierre, Strasbourg, France; 13 Pediatric Nephrology, 295 Bd Abdelmoumen, Casablanca, Morocco; 14 Kidney Hospital, Damascus, Syria; 15 Department of Nephrology, Jundishapur University of Medical Sciences, Ahvaz, Iran; 16 Hospital de Ninos Ludovica La plata, La plata, Argentina; 17 Department of Pediatrics of Gulhane Military, Gulhane, Turkey; 18 Division of nephrology, Children’s hospital of Eastern Ontario, Ottawa, Canada; 19 Centre de référence des maladies rénales rares - Néphrogones, Hôpital Femme–Mère-Enfant, 59, boulevard Pinel, 69677 Bron cedex, France

**Keywords:** Nephropathic cystinosis, Cysteamine, Developing nations

## Abstract

**Background:**

Nephropathic cystinosis is a rare inherited metabolic disorder leading to progressive renal failure and extra-renal comorbidity. The prognosis relies on early adherence to cysteamine treatment and symptomatic therapies. Developing nations [DiN] experience many challenges for management of cystinosis. The aim of this study was to assess the management characteristics in DiN compared with developed nations [DeN].

**Methods:**

A questionnaire was sent between April 2010 and May 2011 to 87 members of the International Pediatric Nephrology Association, in 50 countries.

**Results:**

A total of 213 patients were included from 41 centres in 30 nations (109 from 17 DiN and 104 from 13 DeN). 7% of DiN patients died at a median age of 5 years whereas no death was observed in DeN. DiN patients were older at the time of diagnosis. In DiN, leukocyte cystine measurement was only available in selected cases for diagnosis but never for continuous monitoring. More patients had reached end-stage renal disease in DiN (53.2 vs. 37.9%, *p* = 0.03), within a shorter time of evolution (8 vs. 10 yrs., *p* = 0.0008). The earlier the cysteamine treatment, the better the renal outcome, since the median renal survival increased up to 16.1 [12.5−/] yrs. in patients from DeN treated before the age of 2.5 years of age (*p* = 0.0001). However, the renal survival was not statistically different between DeN and DiN when patients initiated cysteamine after 2.5 years of age. The number of transplantations and the time from onset of ESRD to transplantation were not different in DeN and DiN. More patients were kept under maintenance dialysis in DiN (26% vs.19%, *p* = 0.02); 79% of patients from DiN vs. 45% in DeN underwent peritoneal dialysis.

**Conclusions:**

Major discrepancies between DiN and DeN in the management of nephropathic cystinosis remain a current concern for many patients living in countries with limited financial resources.

**Electronic supplementary material:**

The online version of this article (doi:10.1186/s12882-017-0633-3) contains supplementary material, which is available to authorized users.

## Background

Nephropathic cystinosis is an orphan autosomal recessive lysosomal storage disease characterized by a deficiency of the cystine lysosomal transport protein, cystinosin that is encoded by the *CTNS* gene [1–3]. This is responsible for systemic accumulation of cystine crystals, thus leading to tissue damage, primarily in the kidney and the cornea. The estimated incidence is 1 out of 100,000 to 200,000 living births in developed nations [DeN]. At early stages of the disease, patients suffer from complete proximal tubulopathy in the typical form; the natural evolution of renal damage is a progressive chronic interstitial nephritis, leading to end-stage renal disease [ESRD] in the first decade of life [4–9]. Since the 80’s, cysteamine therapy has postponed ESRD and other extrarenal morbidities to the second (sometimes even the third) decade of life in DeN [1, 9–16]. In developing nations [DiN] that have to face many global challenges when treating children with complex, rare and chronic kidney diseases [CKD], very few data are available on treatment accessibility, growth and renal outcomes in patients with cystinosis [17–24]. We recently demonstrated the existence of major discrepancies between DiN and DeN for renal survival, growth and treatment accessibility [25]. The aim of this paper is therefore to present the complete results obtained from a world-wide multicentre survey reporting the main challenges in the management of cystinosis and comparing outcomes between DiN and in DeN.

## Methods

A questionnaire including 21 items and 10 open-questions about demographics, renal function, management, as well as clinical and biological monitoring, was sent by e-mail to pediatric nephrology centres (Velibor Tasic, VT and Aurelia Bertholet-Thomas, ABT), members of the International Pediatric Nephrology Association (IPNA) mailing list between April 2010 and February 2011 (Additional file 1). In case of response, in each centre, one physician sent back the questionnaire to VT or ABT. The study was made between 2010 and 2011. At the time of the questionnaire, this anonymized retrospective survey did not require an approval of the Institutional Review Board in France, but was secondary approved (Hospices Civils de Lyon IRB, 1/23/2017).

DiN and DeN were defined according to the International Monetary Fund. The diagnosis of nephropathic cystinosis was based on DNA study; if genotyping was not available, the diagnosis was based on the association of renal Fanconi syndrome and corneal crystals deposits on slit lamp eye examination. Demographics included gender, age at diagnosis, as well as age, height [Ht] and body weight [BW] at the last follow-up. Ht and BW were expressed as standard deviation score [SDS] referring to French growth charts [26]. The glomerular filtration rate [GFR] was estimated from serum creatinine using the 2009-revised Schwartz formula [27]. We collected GFR at the last visit, age at CKD5 (i.e., GFR below 15 mL/min per 1.73 m^2^), as well as age at the time of renal replacement therapy [RRT]. For oral cysteamine treatment, we recorded its availability, the age at beginning and the daily current dose (mg/m^2^). Taking other treatments during follow-up such as indomethacin, recombinant human growth hormone [rhGH] and/or nutritional support was recorded. Regarding ophthalmological care, we recorded the results from slit lamp eye examination, the availability of cysteamine eye drops and the median number of drops per eye per day at last visit. We also recorded the distance from home to the medical centre, as well as the number of visits per year with a pediatric nephrologist. The specific biological follow-up was based on white blood cell [WBC] cystine levels, so that the availability of the assay was recorded, as well as the last available half-cystine level (nmol/mg protein) per patient and the number of measurements per year.

For statistical analysis, categorical variables were expressed as number [N] and percentage. The hypothesis of normal distribution of quantitative variables was tested using the Kolmogorov-Smirnov test. Quantitative variables were expressed as means ± standard deviation [SD] when the distribution was normal or median [minimum–maximum] in case of skewed distribution. Categorical variables were compared using the Chi 2 test or Fisher’s exact test when the conditions of application of Chi square test were not met. Quantitative variables were compared between groups using Student’s t test after verification of equality of variances when data were normally distributed, and with the nonparametric Wilcoxon test statistics when the hypothesis of normality of distribution was not verified. Time to CKD5 was calculated from the date of diagnosis or date of cysteamine treatment to the date of CKD5 or the date of the last visit. Survival curves were obtained with a Kaplan-Meier model and groups were compared using the Log Rank test. Missing data were verified but not imputation procedure was conducted.

The statistical tests were bilateral and the level of significance was set to 5% (*p* < 0.05). Statistical analyses were conducted using SAS version 9.3 (SAS Institute Inc., NC).

## Results

### Participating countries

Eighty-seven centres had been approached from all continents and 41/87 reported a total of 213 patients, 109 from DiN and 104 from DeN (Table 1). Of note, 23 other DiN centres answered, while declaring the absence of cystinosis patients.

**Table 1:** Number of patients depending on the nation of origin

Developing nations		Developed nations	
Name	*N*	Name	*N*
Algeria	1	Belgium	5
Argentina	11	Canada	6
Armenia	1	Greece	1
Brazil	13	Czech Republic	1
Chile	2	Finland	1
India	10	France	31
Iran	28	Germany	14
Jordan	5	Israel	2
Lebanon	1	Italy	23
Lithuania	1	Japan	4
Morocco	6	South Korea	1
Poland	2	Spain	5
Russia	7	UK	10
Serbia	3		
South Africa	1		
Syria	6		
Turkey	11		
Total	109		104

### Demographics and treatments

Table 2 summarizes clinical and therapeutic data in both DiN and DeN. Eight out of the 109 patients (7.3%) from DiN died at a median age of 5.0 [0.5–17.7] years, whereas all 104 patients from DeN were alive by the end of follow-up. DiN patients were slightly older at the time of diagnosis compared to DeN patients, i.e., 1.5 [0.0–33.0] vs.1.3 [0.0–9.7] yrs. (*p* = 0.04), respectively; however, the proportion of patients with a diagnosis before 2 years of age was not different between the two groups. Despite the inclusion criterion that was “infantile form of nephropathic cystinosis”, two patients probably rather presented with juvenile forms of cystinosis since they were diagnosed at 16 and 33 years. Unfortunately, more details on these two cases were not available.

**Table 2:** Results

Variables	Developing nations		Developed nations		*p*
	*N*	Median [min-max]/*N* (%)	*N*	Median [min-max]/*N* (%)	
Demographic features	109		104		
Sex ratio (M/F)	84	42/42 (50.0/50.0)	103	52/51 (50.5/49.5)	0.94^c^
Age at diagnosis (yrs)	108	1.5 [0.0–33.0]	104	1.3 [0–9.7]	0.04^a^
Follow-up until diagnosis	108	4.55 [0.0–26.2]	104	5 [0.75–33.6]	0.02^a^
Age at last visit (yrs)	109	8.0 [0.5–35.0]	103	13 [1.2–38.6]	0.001^a^
Diagnosis before 2 yrs. of age	108	63 (58.3)	103	68 (66.0)	0.25
SD weight	77	−3.6 [−9.3–2.3]	101	−0.8 [−6.4–4.2]	<0.0001^b^
SD height	75	−3.5 [−11.7–1.7]	100	−1.58 [−7.5–1.8]	<0.0001^a^
*CTNS* mutation analysis	102	23 (22.6)	97	68 (70.1)	<0.0001^c^
Distance centre – home (km)	68	60 [1–1000]	91	50 [0–20,000]	0.11^a^
Number of visit	99	5 [4–7]	97	4 [4–6]	0.74^a^
Cysteamine Treatment					
Patient treated	106	57 (53.8)	101	101 (100.0)	<0.0001^c^
Age at treatment (yrs)	57	2.0 [0.0–33.0]	100	1.5 [0.0–12.0]	0.02^a^
Patients treated before 2.5 years of age	57	29 (50.9)	100	73 (73.0)	0.005^c^
Median dose (mg/m^2^ per day)	53	1050 [100–1800]	98	1265 [157–2769]	0.0002^a^
Other treatments					
Indomethacin	95	13 (13.7)	100	58 (58.0)	<0.0001^c^
rhGH	95	10 (10.5)	100	56 (56.0)	<0.0001^c^
Tube feeding	95	2 (2.1)	102	27 (26.5)	<0.0001^d^
Cysteamine eye drops	105	22 (20.9)	98	87 (88.8)	<0.0001^c^
Number of drops per eye per day	22	6 [1–14]	91	4 [0–20]	0.05^a^
Renal follow up					
ESRD	109	58 (53.2)	103	39 (37.9)	0.03^c^
Age at ESRD	58	8.0 [0.5–17.5]	35	10·0 [4.0–19.5]	0.0008^a^
Transplanted patients	56	34 (60.7)	38	31 (81.6)	0.86^c^
Delay between ESRD and graft (yrs)	34	2 [0–14]	28	1 [0–11]	0.25^a^
Patients on dialysis (PD/HD)	28	22/6	20	9/11	0.02

*SD* standard deviation, *ESRD* end stage renal disease, *PD* peritoneal dialysis, *HD* hemodialysis

^a^Wilcoxon test

^b^Student test

^c^Chi-square test

^d^Fisher test

The *CTNS* mutation analysis was performed in 23% vs. 70% of patients in DiN and DeN respectively (*p* < 0.001). When genotyping was not available other techniques were used, such as slit lamp eye examination in 87% of cases. The number of visits per year and the distance from home to reference centre were not different between the two groups. The farther the centre in DiN, the lower the number of visits per year (*r* = −0.33, *p* = 0.002).

Patients receiving cysteamine in DiN were significantly older at treatment initiation, 2 [0.0–33.0] vs. 1.5 [0.0–12.0] yrs. (*p* = 0.02). Oral cysteamine was started before 2.5 years of age in 51% of patients from DiN and in 73% of patients from DeN (*p* = 0.005); however, when available, cysteamine was initiated during the first month after diagnosis in all patients. The median cysteamine dose was 1050 [100–1800] and 1265 [157–2769] mg/m^2^ per day in DiN and DeN, respectively (*p* = 0.0002). The cost of cysteamine bitartrate ranged between 11.4 € and 47.5 € per gram in 9 of the 17 DiN. WBC cystine level was available for 94% of patients treated in DeN (*N* = 91), and their last median half-cystine level was 1.1 [0.2–12] nmol/mg protein. The median number of dosages per patient and per year was 4 [1–6] (*N* = 71). In DiN, WBC cystine level was only mentioned for two patients in whom it was used for diagnosis but not to adapt the dose as recommended.

Few patients from DiN received adjunctive therapies such as indomethacin, rhGH or enteral nutritional support (respectively 14, 11 and 2%) whereas 56% patients from DeN received rhGH, 58% indomethacin and 27% received enteral nutritional support. In the DeN group, there was no significant difference for mean growth velocity (SDS height and weight) at last examination between groups of patients using or not adjunctive treatments (*p* = 0.06 and *p* = 0.15, respectively).

Cysteamine eye drops were given only to 21% of patients in DiN vs. 89% in DeN (*p* < 0.0001). In addition, the number of cysteamine eye drops per eye per day was 6 [1–14] in patients from the DeN vs. 4 [0–20] in patients from DiN (*p* = 0.05).

### Evolution of renal function

At the last follow-up, more patients from DiN reached ESRD than those from DeN: 53% vs. 38%, respectively (*p* = 0.03); the onset of ESRD occurred earlier in DiN, at a median age of 8.0 [0.5–17.5] vs. 10 [4–19.5] yrs. in DeN (*p* = 0.0008). The median renal survival in DiN was two-time shorter than in DeN, i.e., 6.3 [5.3–8.4] vs. 12.7 [11.2−/] yrs., respectively (*p* < 0.0001).

**Fig. 1:**
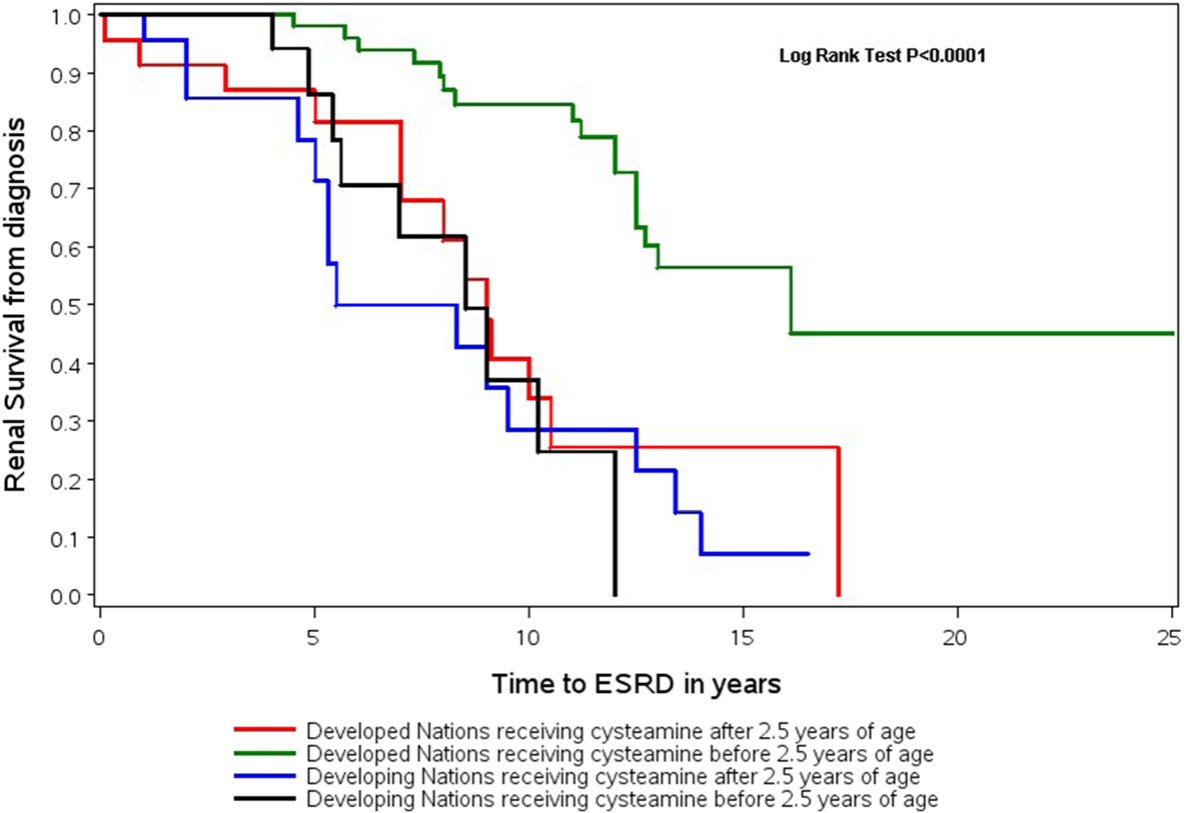
Renal survival depending on age initiation of cysteamine and country of origin

Moreover, the earlier the cysteamine treatment, the better the renal outcome, since the median renal survival increased up to 16.1 [12.5−/] yrs. in patients from DeN treated before the age of 2.5 years of age (*p* = 0.0001), as shown in Fig. 1. However, in DiN, there were no statistical differences in renal survival between patients starting cysteamine therapy before 2.5 and after 2.5 years of age (*p* = 0.47). The renal survival was not statistically different between DeN and DiN when patients initiated cysteamine after 2.5 years of age.

Although there was no difference between growth and associated treatments, renal survival was significantly improved, both in DiN and DeN, among patients using adjunctive treatments compared to patients not using adjunctive treatments (*p* = 0.004 and *p* = 0.03, respectively).

The proportion of transplanted patients was 61% in DiN vs. 82% in DeN (*p* = 0.87). The delay between ESRD and transplantation was 2 years in DiN and 1 year in DeN (*p* = 0.25). More patients were kept under maintenance dialysis in DiN, i.e., 26% vs.19% in DeN (*p* = 0.02); 79% of patients from DiN vs. 45% in DeN underwent peritoneal dialysis.

Table 2 summarizes renal outcomes in the two groups.

## Discussion

This study suggests remarkable discrepancies in the management of patients with nephropathic cystinosis throughout the world, with a comparison between DiN and DeN including 213 patients from 41 different centres and 30 nations. Although the treatment of nephropathic cystinosis is now well established in DeN, aiming at delaying ESRD and preventing late systemic complications mainly with the new ‘easier-to-take’ oral formulation and ocular cysteamine [28–31], cystinosis is still responsible for a high morbidity and mortality in children and young adults living in DiN [25].

The current condition of patients with nephropathic cystinosis living in DiN looks like DeN condition during the early 80’s in terms of life expectancy, growth and renal survival [1, 5–9]. Such discrepancies are mainly explained by the financial cost of cysteamine therapy, 1 g cysteamine bitartrate ranging from 11.4 to 47.5 Euros, thus making it less accessible to patients in DiN. As shown in this study, most centres from DiN reported different reasons for the lack of availability to cysteamine, such as cysteamine not available, too expensive, unlicensed, or unacceptable in the context of national priorities. In Egypt, Soliman et al. previously estimated a 380 Euros monthly cost of oral cysteamine bitartrate at a low dose of 50 mg/kg per day for a child weighing 15 kg, compared to 280 Euros in France; oral cysteamine was not routinely available in Egypt [20]. Some patients or medical staff had arranged illegal supplying traffic: cysteamine was bought in Europe or North America by family friends or physicians during conferences and brung back to their native countries. Cysteamine eye drops were regarded as a minor issue in DiN centres and were not available in most of them, so that only 21% of patients could benefit from it in contrast to 89% of patients in DeN.

We had also hypothesized a difference in medical education between DiN and DeN, and Public Health priorities are different from one country to the other. There were no significant differences in the quality of medical follow-up: indeed, number of visits per year and distances between care unit and home were not different between patient from DiN and DeN. However, we may speculate that the access to centres was different according to each country but this data was not available for analysis. Moreover, even though it was statistically significant, the difference of age at diagnosis was not clinically relevant, since most patients were diagnosed in the same age range whatever the method used (DNA analysis and/or WBC cystine levels in DeN, search for corneal crystals in DiN). Eventually, the quality of data collection was conducted according to the same process for all responding centres.

There is very little published data on cystinosis originating from DiN. Vaisbich et al. reported clinical outcomes in 102 cystinotic patients in Brazil, highlighting late diagnosis, high mortality rate (10%), and poor growth, as only 15% of the patients could receive rhGH. In this cohort, 59% of patients were in CKD5. There was no national supply for cysteamine and WBC cystine level measurement was not available; patients bought cysteamine eye drops by their own [17]. Al-Nabhani et al. published in 2011 the first reported case of a 21 month-old cystinotic patient in Oman [18, 19].

In Egypt, Soliman et al. reported a 80% consanguinity rate in their cohort of 33 patients; 70% of them were diagnosed before 2 years of age, usually using slit-lamp eye examination [20]. Untreated patients reached ESRD before the age of 10 years. Despite under-diagnosis and under-treatment, the authors hypothesized that different phenotypes could be associated with different genotypes in the world [20–22]. In this questionnaire, we could hypothesize that the high mortality rate observed in DiN in comparison to DeN could at least partly be explained by different genotype-phenotype correlations, in addition to likely under-diagnosis and poor cysteamine access.

Most of our DeN data come from European centres that could explain why our results are not different from those published by Greco et al. describing the renal survival in 23 Italians patients followed for 5 years [11]. In this paper, most patients had reached CKD3 at 10 years of age, but all patients treated before 2.5 years of age had a longer renal survival. Brodin-Sartorius et al. reported on a French cohort of 86 adult cystinotic patients diagnosed between 1961 and 1995 also with a median age at ESRD of 9.9 yrs. [32]. However in patients treated before 5 years of age, the median age at ESRD was 12.2 yrs., compared to a median age of 16 yrs. for patients treated before 2.5 yrs. of age in DeN. This difference could be explained by the improvement of medical care during the past 20 years in DeN and by the decrease of the optimal age for starting cysteamine treatment from 5 to 2.5 yrs. Thus, the age at initiation of cysteamine therapy appears to be a major predictive factor of renal survival. Other treatments such as indomethacin, enteral nutritional support, or rhGH also influence the renal prognosis: the more active adjunctive therapies, the better renal survival [33–35]. It is difficult to decide whether this combination of therapies can directly explain a better renal survival or whether these therapies are only an indirect marker of a closer follow-up, since adherence to treatment is an important issue regarding morbidity in DeN, and is specifically critical in teenagers. Ariceta et al. reported that children older than 11 years of age and adults were dramatically less compliant, only half of them reporting to always follow prescription [36]; as such, these authors proposed recommendations for a comprehensive care of nephropathic cystinosis [37].

However, a minority of patients from DiN could benefit from adjunctive measures that may, at least partly, explain the absence of correlation between renal survival and age at the start of cysteamine therapy in our study population.

In the early 2000’s, Gahl and Schneider suggested that early cysteamine therapy with both optimal adherence and adequate WBC cystine levels could prevent systemic morbidity, and notably the renal ones [1, 10, 14]. However, renal tubular dysfunction persists, glomerular filtration rate decreases and late complications appear despite cysteamine treatment. Schneider therefore summarized the management of cystinosis as ‘simple in principle but difficult in practice’ [38]. More than 62% of patients are commonly non-compliant. Brodin-Sartorius et al. reported a ‘quite good’ compliance rate for 76% of patients but 30% of them presented extended periods without treatment [32]. Despite these difficulties in terms of treatment management and adherence, our data showed that cysteamine therapy improves renal survival from 3.8 yrs. in untreated patient from DiN to 16 yrs. in well treated patients from DeN. Furthermore, Van Stralen et al. provided an analysis of 134 cystinotic patients from Western European countries from the ESPN/ERA-EDTA registry: between 1979 and 2008, the mean age at start of RRT increased by 0.15 year per calendar year from 8.8 to 12.7 years, whereas it was not observed for non-cystinotic children. They hypothesized that it was a direct consequence of an earlier initiation of cysteamine therapy during this study period [39].

In the future, the gap between DiN and DeN may hopefully become smaller, if DiN catch up with DeN standards: it has been the case for example in Turkey in the recent years. However, this adaptation will take a different time depending on the countries. Some factors will shorten the process of adaptation to high standards such as a good training of young pediatric nephrologists, the World Kidney Day initiative, workshops and patients’ days focused on inherited kidney diseases in some DiN (e.g., in Egypt or Iran), whereas others will obviously delay the adaptive process. For example, the high annual cost of the long-lasting cysteamine therapy (that is currently progressively available in DeN) will hinder DiN from catching up with DeN, because renal survival will probably further improve in DeN by reducing non-compliance. It is crucial that all DiN nations can access cysteamine, but other factors can also be identified to improve the quality of care of cystinosis in DiN: for example, companies could try to develop cheaper tools for early diagnosis of tubulopathy, namely by developing a less expensive urinary dipstick that would only detect proteinuria, hematuria and glycosuria. We could hope that new emerging approaches to treat cystinosis such as new galenic forms, novel compounds and hematopoietic stem cells transplantation will delay the onset of ESRD and thus improve the global survival of patients in DeN but also in DIN [40–42].

In addition to a reinforced and focused teaching of early identification of tubulopathies and clinical phenotypes of nephropathic cystinosis to paediatricians, this possibility to provide a cheap urinary stick worldwide would have a great impact to improve the diagnosis of renal diseases (and therefore their early management) in DiN. Last, in addition to a late diagnosis, one of the main obstacles for improving renal care of children is probably the inequity of care in DiN, adult patients likely receiving a better care than children. In this setting, the 2016 international World Kidney Day was directly directed towards pediatric CKD: ‘Act early to prevent it!’, and many initiatives have been undertaken in DiN to enhance the knowledge and awareness of pediatric CKD worldwide.

## Conclusion

Our study has some limitations. The retrospective survey-based data collection could lead to information, and measurement biases, and as our survey data did not originate from an international registry and did not cover the US, the potential for selection bias exists. It is not clear whether data from North America would have changed the results we observed between DiN and DeN. Moreover, the self-reporting could also create a bias in the results as we could not be sure that survey responders were representative for their own countries. It would also have been interesting to evaluate whether there were differences between larger and smaller centres (in terms of number of patients under care); however, we did not have enough statistical power to perform such an analysis. This survey remains, however, an appropriate and acceptable method for a first global description of the clinical management of cystinosis, an orphan disease.

In conclusion, as for many orphan pediatric diseases [43], major discrepancies between DiN and DeN in the management of nephropathic cystinosis remain a critical concern for many patients, mainly due to different access to available specific drugs. International networks and registries for orphan diseases are an important tool for reducing health inequalities between developed and developing countries.

## Additional file

Additional file 1: The copy of the questionnaire that was sent by mail. (DOCX 12 kb)

## Abbreviations

ABT: Aurelia Bertholet-Thomas
BW: Body weight
CKD: Chronic kidney disease
DeN: Developed countries
DiN: Developing countries
ESRD: End-stage renal disease
GFR: Glomerular filtration rate
Ht: Height
IPNA: International Pediatric Nephrology Association
N: Number
rhGH: Recombinant human growth hormone
RRT: Renal replacement therapy
SD: Standard deviation
SDS: Standard deviation score
VT: Velibor Tasic
WBC: White blood cell
