# Supplementary material for: Worldwide view of nephropathic cystinosis: results from a survey from 30 countries
Source: BMC Nephrol. 2017 Jul 3;18:210. doi: 10.1186/s12882-017-0633-3 (PMC5496396; doi:10.1186/s12882-017-0633-3)
Supplement: Additional file 1: — The copy of the questionnaire that was sent by mail. (DOCX 12 kb) [file 12882_2017_633_MOESM1_ESM.docx]

**Additional file: the copy of the questionnaire that was sent by mail**

Centre and country:

Management of cystinosis patients at your centre

Patient No

Gender

Age at diagnosis (yr)

Age at last visit (yr)

Height at last visit (cm, SDS)

BW at last visit (kg, SDS)

DNA study (y/n)

Renal function

Last GFR (Schwartz)

Age at ESRD (yr)

Age at start of dialysis

PD/HD

Age at first renal transplantation (yr)

Cysteamine treatment

Age at beginning of cysteamine treatment (yr)

Current dose of cysteamine (mg/m²/d)

Associated treatment at any time

Indomethacin (y/n)

Growth hormone (y/n)

Tube feeding (y/n)

Eyes

Slit lamp eye examination available (y/n)

Average number of cysteamine eye drops per day (per eye)

Clinical follow up

Distance between medical centre and home (km or hours)

Number of visits per year

Leucocyte cystine assay

Leukocyte cystine assessment available (y/n)

Last half-cystine level (nmol/mg of protein)

Number of half-cystine dosage per year

We will be very obliged if you could answer to this few questions?

1- How many pediatric nephrologists work at your centre?

And in your country?

How do you confirm a hypothesis of cystinosis in your country?

2- Which cysteamine formulation do you use for oral treatment?

What is the cost per 1 g?

For eye drops?

What is the cost per 10 mL?

3- Which company provides you with oral cysteamine?

Which company provides you with eye drops?

4- How is cysteamine delivered to your patients?

5- Which technique is used at your centre for leukocyte cystine assay?

Could you describe it shortly?

Which target do you use under cysteamine treatment?

6- Which laboratory performs this dosage?

Is it a private lab or a national/public one?

And how far is it from our centre?

What is the cost of the assay?

How do you ship blood samples?

7- How is the health care system in your country: public/state healthcare? private insurance? Both?

8- Which parameters do you use for cystinosis patient monitoring?

9- Could you summarize the main difficulties for managing cystinosis patient in your centre?

10- On your opinion, what would improve such a condition?

Free comments about cystinosis at your centre:
